# Enhanced Electrochemical Performance of Dual-Ion Batteries with T-Nb_2_O_5_/Nitrogen-Doped Three-Dimensional Porous Carbon Composites

**DOI:** 10.3390/molecules30020227

**Published:** 2025-01-08

**Authors:** Chen Qi, Duo Ying, Cheng Ma, Wenming Qiao, Jitong Wang, Licheng Ling

**Affiliations:** 1 State Key Laboratory of Chemical Engineering, East China University of Science and Technology, Shanghai 200237, China; 18919095863@163.com (C.Q.); shapeofvoice@163.com (D.Y.); mac@ecust.edu.cn (C.M.); qiaowm@ecust.edu.cn (W.Q.); lchling@ecust.edu.cn (L.L.); 2 Guangxi Key Laboratory of Petrochemical Resource Processing and Process Intensification Technology, School of Chemistry and Chemical Engineering, Guangxi University, Nanning 530004, China

**Keywords:** dual-ion batteries, Nb_2_O_5_, anode, structure design, Nitrogen doping

## Abstract

Niobium pentoxide (T-Nb_2_O_5_) is a promising anode material for dual-ion batteries due to its high lithium capacity and fast ion storage and release mechanism. However, T-Nb_2_O_5_ suffers from the disadvantages of poor electrical conductivity and fast cycling capacity decay. Herein, a nitrogen-doped three-dimensional porous carbon (RMF) was prepared for loading niobium pentoxide to construct a composite system with excellent electrochemical performance. The obtained T-Nb_2_O_5_/RMF composites have a well-developed pore structure and a high specific surface area of 1568.5 m^2^ g^−1^, which could effectively increase the contact area between the material and electrolyte, improving the electrode reaction and lithium-ion transfer diffusion. Nitrogen doping increased surface polarity, creating more active sites and accelerating the electrode reaction rate. The introduction of T-Nb_2_O_5_ imparted high power density and excellent cycling stability to the battery. The composites exhibited good electrochemical performance when used as dual-ion battery anode, with a stable cycle life of 207.2 mA h g^−1^ at 1 A g^−1^ current density after 650 cycles and great rate performance of 181.5 mA h g^−1^ at 5A g^−1^ was also obtained. This work provides the possibility for applying T-Nb_2_O_5_/RMF as an anode for a high-performance dual-ion battery.

## 1. Introduction

The current depletion of fossil fuel reserves and the growing problem of environmental pollution have prompted extensive research into the development and storage of new green energy sources [1,2]. The intermittent nature of renewable solar and wind energy necessitates the development of energy storage solutions [3,4]. Lithium-ion batteries have gained widespread commercial applications due to their good energy storage performance and stability [5]. However, limited and unsustainable lithium and cobalt resources, high costs and safety concerns remain [6,7,8,9,10]. Consequently, it is essential to investigate new electrochemical energy storage devices that offer high safety, low cost, and environmental sustainability, as well as high energy density and long cycle life.

Dual-ion batteries (DIBs) represent a promising class of energy storage technologies, distinct from traditional alkali-metal ion batteries that operate on the ‘rocking chair’ mechanism [11]. In this case, cations shuttle between the positive and negative electrodes during charging and discharging. In contrast, DIBs involve both cations and anions during charging and discharging. Cations and anions enter the anode and cathode simultaneously during charging, and then released from the electrodes simultaneously during discharging [12,13,14]. This unique mechanism contributes to the high energy density of DIBs, similar to that of lithium-ion batteries, while also offering a higher operating voltage and improved environmental friendliness [15,16]. Consequently, DIBs have become an active area of research in recent years. The electrode material is a crucial factor limiting the development of DIBs, with the anode material typically responsible for the intercalation and deintercalation of cations. Graphite is commonly utilized as the anode material in DIBs due to its layered structure, which facilitates the reversible intercalation of various cations. While graphite helps reduce the overall cost of DIBs, it has limitations in terms of lithium intercalation capacity and cycling stability. Specifically, graphite has a relatively low lithium intercalation capacity of 372 mAh g^−1^ and a low intercalation potential (~0.1 V vs. Li/Li^+^), which is close to the lithium deposition potential. This proximity increases the risk of dendrite formation during cycling, which can compromise battery safety by causing short circuits [17,18]. Furthermore, the cycling stability of graphite is also a concern, as the structural integrity of its layered framework is prone to degradation during the insertion and extraction of lithium ions [19,20]. Therefore, in this work, it is desired to prepare a DIBs anode material to improve the lithium intercalation capacity, cycling stability and structural recovery.

The anode ion storage mechanism of dual-ion batteries is similar to that of lithium-ion batteries [21], and is also divided into intercalation [22,23], alloying [23,24], conversion [25,26], and organic [27,28] anodes materials [29]. For dual-ion batteries, an ideal anode material should demonstrate a favorable cation reduction reaction and rapid ion transport kinetics, aligning with the swift insertion kinetics of anions on the cathode side to maintain robust electrochemical performance, even under high current densities [30,31,32]. Additionally, the anode material must exhibit a strong binding capacity to prevent the spontaneous release of active ions, thereby reducing the self-discharge rate [33]. Good compatibility with the electrolyte is also necessary, allowing for the rapid formation of a solid electrolyte interface (SEI) that enhances cation transport efficiency and extends the electrode’s operational lifespan [34,35]. The electrolyte can be stabilized and concentrated under high pressure. The orthorhombic crystal system of Nb_2_O_5_ (T-Nb_2_O_5_) has been identified as a conversion anode with a unique pseudo-capacitance effect [36,37]. The insertion and detachment of lithium ions in this material are characterized by a fast charge storage process, which can produce high energy density without phase transition [38]. Nevertheless, it is important to acknowledge that pure Nb_2_O_5_ also exhibits certain limitations as an anode material. These include large potential lag during cycling, low initial cycling coulombic efficiency, rapid capacity decay, and poor rate performance [36,39,40]. To compensate for these problems, Nb_2_O_5_-based nanocomposites require more conductive networks to achieve higher performance.

Based on these, in this work, T-Nb_2_O_5_/nitrogen-doped three-dimensional porous carbon composites were synthesized by in-situ growth of T-Nb_2_O_5_ nanoparticles on the surface and inside the pores of a nitrogen-doped mesoporous carbon (RMF). The loading could be easily controlled by adjusting the ratio of ammonium niobium oxalate and RMF. The nitrogen-doped mesoporous carbon has a highly developed pore structure, which possesses a large amount of space for the embedding of T-Nb_2_O_5_. The constructed three-dimensional mesh skeleton provides excellent structural stability. Meanwhile, the growth of T-Nb_2_O_5_ in the pore channels makes the larger pores into micropores with smaller pore diameters and larger specific surface area, which can effectively increase the contact area between the material and electrolyte. This is conducive to the electrode reaction and lithium-ion transfer diffusion at the electrode interface. Meanwhile, the doping of nitrogen breaks the uniform arrangement of electrons on the surface of the carbon structure, which enhances the surface polarity of the material, helps the conduction of charge in the composite structure, and accelerates the reaction kinetics. In addition, T-Nb_2_O_5_ has a unique pseudocapacitance property that provides a high-speed ion embedding mechanism and good electrochemical stability. Therefore, the dual-ion battery with T-Nb_2_O_5_/RMF as the anode was able to achieve an initial specific capacity of 332.8 mA h g^−1^ at a high current density of 1 C, and still retained a specific capacity of 207.2 mA h g^−1^ after 650 cycles, with a decay rate of only 0.058% per cycle. The T-Nb_2_O_5_/RMF composites prepared in this work are able to achieve high electrochemical performance as a simple and effective strategy for the practical application of dual-ion battery anodes.

## 2. Results and Discussion

### 2.1. Synthesis and Characterization of Materials

**Figure 1:**
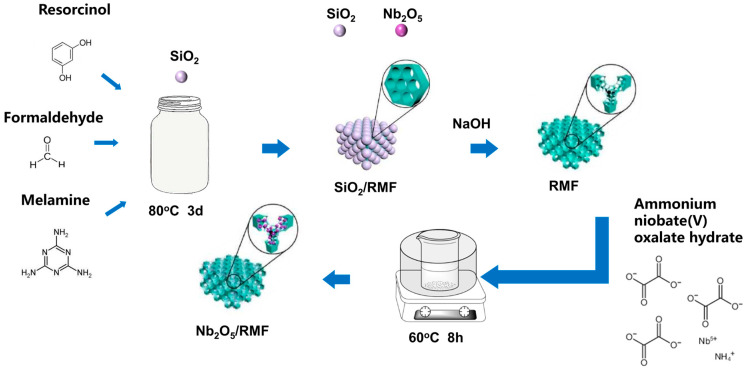
Synthesis route of Nb_2_O_5_/RMF.

The approximate synthetic route of T-Nb_2_O_5_/RMF is shown in Figure 1. The RMF was synthesized using SiO_2_ as a template, resorcinol-formaldehyde resin as a carbon source, and melamine as a nitrogen source. Subsequently, T-Nb_2_O_5_ was loaded on the RMF by a simple water bath using niobium ammonium oxalate as a niobium source. A series of materials were prepared by varying the ratios of ammonium niobium oxalate and RMF. As a comparison, in addition to T-Nb_2_O_5_/RMF loaded with T-Nb_2_O_5_ on 3D porous carbon RMF, T-Nb_2_O_5_/CNTs loaded with T-Nb_2_O_5_ on 1D carbon nanotubes and T-Nb_2_O_5_/GO loaded with T-Nb_2_O_5_ on 2D graphene were also prepared in this work. The synthesized three-dimensional porous carbon RMF has a high specific surface area and abundant pore structure, which can effectively load T-Nb_2_O_5_. Nitrogen doping destroys the surface polarity of the carbon material, which makes it exhibit more active sites and accelerates the electrode reaction rate. The T-Nb_2_O_5_/RMF loaded with T-Nb_2_O_5_ has a higher specific surface area and a developed porechannel structure, which provides a place for lithium-ion storage and transport and improves the battery performance.

**Figure 2:**
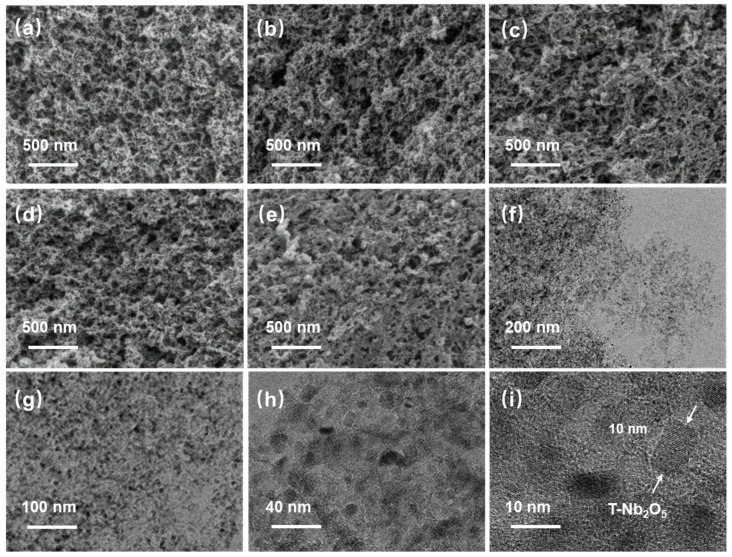
SEM images of (**a**) RMF, (**b**) T-Nb_2_O_5_/RMF 1:1, (**c**) T-Nb_2_O_5_/RMF 1:2, (**d**) T-Nb_2_O_5_/RMF 1:6, (**e**) T-Nb_2_O_5_/RMF 1:10, and (**f**–**i**) TEM images of T-Nb_2_O_5_/RMF 1:6.

In order to clearly observe the microscopic morphology of the samples in different scales and the dispersion state of T-Nb_2_O_5_ in the mesoporous carbon materials. The scanning electron micrographs (SEM) and transmission electron micrographs (TEM) were taken for the samples. As can be seen from Figure 2a, the RMFs prepared after selective etching of the silicon templates with NaOH solution show a three-dimensional reticulated structure with fairly well-developed pores. Through Figure 2b–e, it can be found that the pore size of the material tends to decrease gradually with the increasing proportion of niobium source added, which is due to the fact that the addition of more T-Nb_2_O_5_ fills up the pores with larger internal diameters of the carbon material. This also shows that T-Nb_2_O_5_ can effectively grow on the surface and inside the pores of the mesoporous carbon material. Further observation of the micromorphology by transmission electron microscopy in Figure 2f–i shows that T-Nb_2_O_5_ nanoparticles can be found to be uniformly dispersed in the composite with a diameter of about 10 nm, and their lattice can be clearly observed. T-Nb_2_O_5_ grows uniformly on the surface and inside the pores of mesoporous carbon, which provides a more stable spatial structure for T-Nb_2_O_5_ nanoparticles. Meanwhile, the three-dimensional reticulated carbon skeleton enhances the charge transfer rate, and the developed pore structure offers a place for the storage and transport of lithium ions, which is conducive to improving the performance and dynamics of the battery. T-Nb_2_O_5_/GO and T-Nb_2_O_5_/CNTs were also characterized by SEM and TEM, as shown in Figures S1 and S2. From Figure S1, the layered structure of graphene and T-Nb_2_O_5_ nanoparticles dispersed on the surface of graphene can be clearly seen. The layered structure of graphene has been curled to a certain extent and even formed a more complex reticular structure. Figure S2 shows that the carbon nanotubes are irregularly interwoven, and the T-Nb_2_O_5_ nanoparticles are bound on the surface and inside of the carbon nanotubes. Thus, the as-prepared T-Nb_2_O_5_/CNTs composites construct a complex interwoven mesh carbon skeleton with carbon nanotubes randomly interwoven.

**Figure 3:**
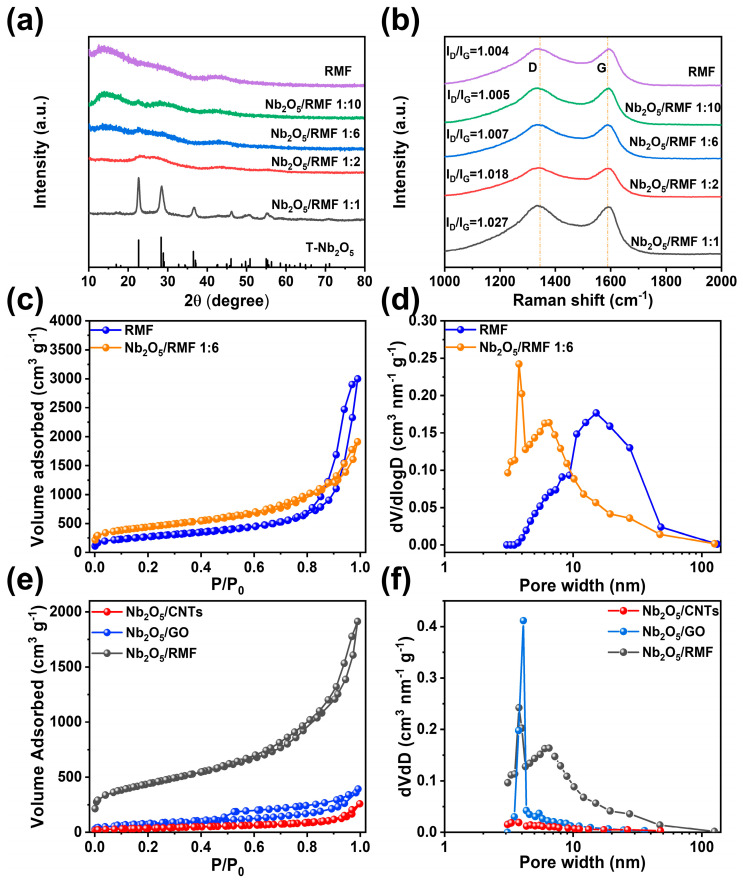
(**a**) XRD pattern (**b**) Raman pattern of composites with different T-Nb_2_O_5_/RMF ratios and RMF. (**c**) Nitrogen isothermal adsorption–desorption curves and (**d**) DFT pore size distribution of T-Nb_2_O_5_/RMF composite with RMF. (**e**) Nitrogen isothermal adsorption–desorption curves and (**f**) DFT pore size distribution of different T-Nb_2_O_5_/C composites.

In order to further analyze the composition and lattice conditions of the composites, the samples with different amounts of T-Nb_2_O_5_ were subjected to XRD and Raman characterization, and the results are shown in Figure 3. Figure 3a presents a comparative XRD analysis of T-Nb_2_O_5_/RMF. The XRD curves of RMF exhibit a wide range of disordered carbon characteristic peaks in the range of 20°–30°, while the diffraction peaks of T-Nb_2_O_5_ gradually become more pronounced with the increase in the introduction of T-Nb_2_O_5_. At a T-Nb_2_O_5_/RMF ratio of 1:1, the characteristic peaks exhibit a clear correspondence with the standard card of T-Nb_2_O_5_ (PDF:30-0873), indicating that T-Nb_2_O_5_ had been effectively synthesized and loaded into the mesoporous carbon material. As illustrated in Figure 3b, the Raman spectra of the composites exhibit two broad carbon peaks, which are the disordered carbon peak (D peak) and the ordered carbon peak (G peak). Generally, the D peak is attributable to the defects resulting from heteroatoms and functional groups, while the G peak is related to the in-plane tensile vibration of sp2-hybridized carbon atoms [41,42]. The intensity ratio of the D peak to the G peak can be employed as an indicator of the degree of defectification of the material surface. The calculation of the peak intensities of the Raman spectra of different materials reveals that the I_D_/I_G_ values of the composites gradually increase with the ratio of T-Nb_2_O_5_/RMF. This indicates that the introduction of T-Nb_2_O_5_ effectively increases the degree of defectivation on the surface of the material. The presence of metal oxides breaks the neatly distributed electronic arrays on the surface of the carbon, enhances the surface polarity of the carbon material, and thus improves the electrical conductivity of the composite material. Figure S3a,b illustrate the XRD and Raman analysis profiles of T-Nb_2_O_5_ when loaded on 1D, 2D, and 3D carbon materials. As illustrated in Figure S3a, the 2D T-Nb_2_O_5_/GO exhibits the most pronounced T-Nb_2_O_5_ characteristic peaks, followed by the 1D T-Nb_2_O_5_/CNTs. However, the T-Nb_2_O_5_/CNTs display the most prominent 26° carbon peaks. Figure S3b illustrates that the 2D T-Nb_2_O_5_/GO has the largest I_D_/I_G_, the 3D T-Nb_2_O_5_/RMF the second largest, and the 1D T-Nb_2_O_5_/CNTs the smallest. It has been demonstrated that an appropriate degree of carbon surface defects enhances the surface polarity of the carbon material and improves the overall electrical conductivity of the composite.

Figure 3c–f demonstrate the nitrogen adsorption/desorption curves with pore size distribution data for the samples. Based on the nitrogen isothermal adsorption/desorption curves of the materials, the specific surface area, total pore volume and average pore diameter of the composites were calculated, as shown in Table 1. It can be seen that RMF exhibits an obvious type IV adsorption/desorption curve and H3 hysteresis loop, indicating that it has a rich mesoporous structure. The specific surface area of RMF is 1008.6 m^2^ g^−1^, and its pore size is mainly distributed around 15 nm, with a pore volume of 4.51 cm^3^ g^−1^. After the introduction of T-Nb_2_O_5_ nanoparticles, the specific surface area of T-Nb_2_O_5_/RMF increased to 1568.5 m^2^ g^−1^, the pore size was mainly distributed around 4 nm, and the pore volume was reduced to 2.79 cm^3^ g^−1^, which may be due to the fact that T-Nb_2_O_5_ nanoparticles split the larger pores into smaller pores with a more complex structure and larger internal surface area. The smaller pores can improve the contact effect between the composite and the electrolyte, as well as the lithium-ion transport rate at the electrode material–electrolyte interface. Comparing the BET data of the T-Nb_2_O_5_ composites with different carbon supports, it can be observed that the specific surface areas show an increasing trend from 1D, 2D to 3D composites, which are 135.3 m^2^ g^−1^, 263.9 m^2^ g^−1^ and 1568.5 m^2^ g^−1^, respectively. The main pore structure size of all three composites is mesoporous, and the pore volume and main pore size distribution also tend to increase with dimension.

**Figure 4:**
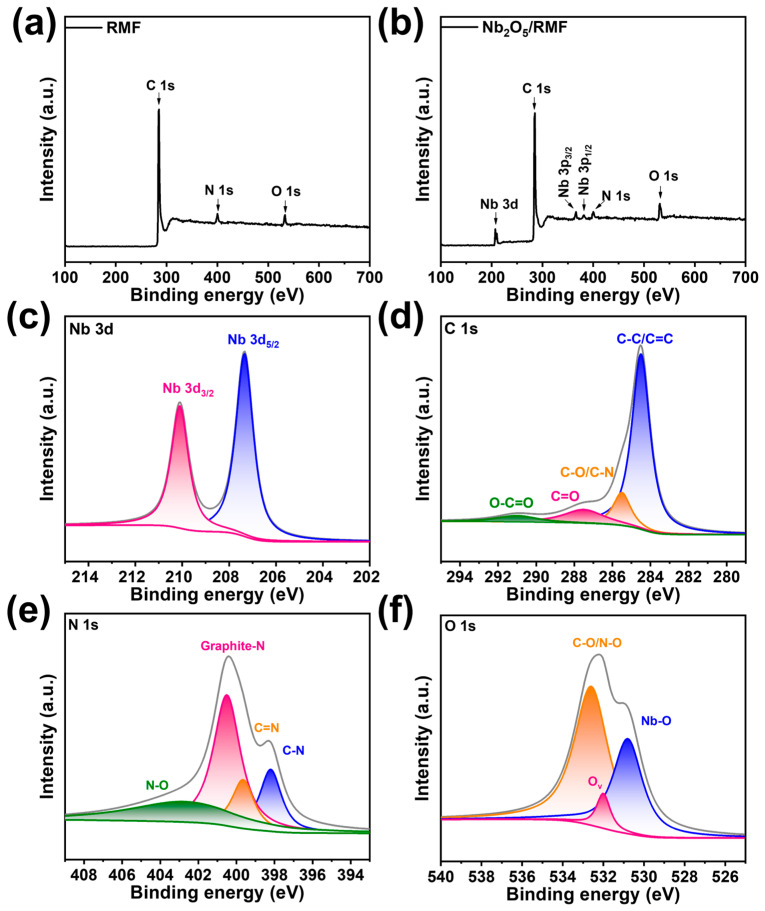
(**a**) XPS full spectral of RMF, (**b**–**f**) XPS spectrum of T-Nb_2_O_5_/RMF composite.

X-ray photoelectron spectroscopy (XPS) characterization determines the elemental composition and chemical bonding energy of the T-Nb_2_O_5_/RMF composites, as shown in Figure 4. XPS analysis reveals the presence of four elements in the composite sample: carbon (C), nitrogen (N), oxygen (O) and niobium (Nb). It can be seen that the C, N and O elements are present in the RMF, and the Nb element is introduced by the compositing process with T-Nb_2_O_5_. By analyzing the fine spectra of the different elements with peak fitting, it is possible to obtain the different valence patterns of the element. As illustrated in Figure 4c for Nb 3d, the Nb 3d spectrum is primarily comprised of two peaks, corresponding to the Nb 3d_5/2_ orbital at 207.3 eV and the Nb 3d_3/2_ orbital at 210.1 eV, respectively. By fitting the analysis in Figure 4d, it can be demonstrated that there are C–C/C=C, C–O, C=C, and C–N bonding modes for the C elements in the material. In Figure 4e, the N 1s are categorized into four forms: N–O, C–N, C=N, and graphitic N. Among these, C–N and C=N confirm that the N element was successfully doped on the surface of the carbon material, which effectively enhances the surface polarity. In the fine spectrum of O 1s, the split-peak fitting yields C–O/N–O, Ov, and Nb–O, corresponding to binding energies at 532.9 eV, 532.1 eV, and 530.8 eV, respectively; Ov represents the presence of oxygen vacancies. Figure S5 demonstrates the XPS spectra of T-Nb_2_O_5_/GO, and Nb_2_O_5_/CNTs; comparing the three composites reveals that T-Nb_2_O_5_/GO has a stronger O peak. This may be due to the presence of oxygen-containing functional groups hydroxyl and epoxy groups in graphene oxide.

In order to accurately determine the mass ratio of carbon to metal elements in the T-Nb_2_O_5_/RMF composites, thermogravimetric analyses were performed in air, and the thermogravimetric data are presented in Figure S4. From the thermogravimetric curves, it is observed that the composites exhibit a significant weight loss at approximately 550 °C. It is also noted that the peak temperature of weight loss increased with the increase in metal content.

### 2.2. Battery Dynamics Analysis

**Figure 5:**
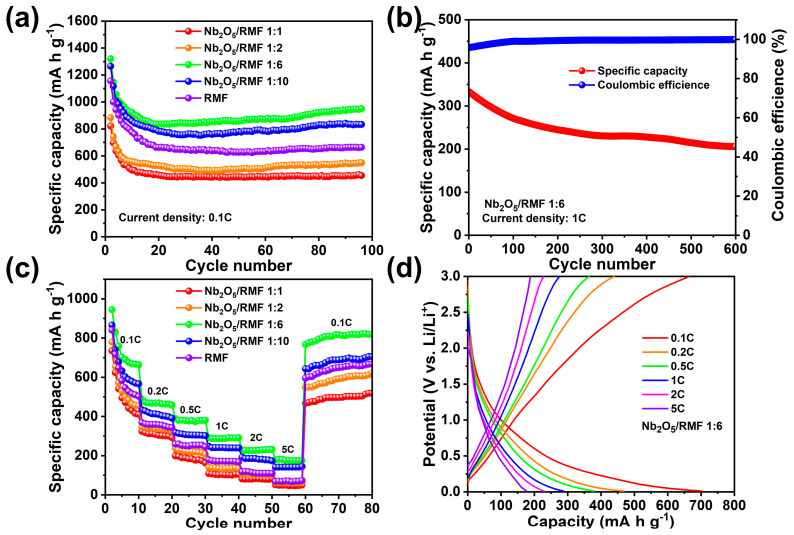
(**a**) Comparison of cycle performance of batteries assembled with different T-Nb_2_O_5_/RMF ratio composites at 0.1 C, (**b**) long cycle performance of batteries assembled with T-Nb_2_O_5_/RMF 1:6 composite at 1 C. (**c**) Comparison of rate performance of batteries assembled with different T-Nb_2_O_5_/RMF ratio composites, (**d**) Rate charge/discharge curves of T-Nb_2_O_5_/RMF 1:6.

In order to investigate the electrochemical performance of the T-Nb_2_O_5_ composite electrode, a half-cell was prepared using a 2025 button cell with lithium metal as the counter electrode and reference electrode. As illustrated in Figure 5a, the electrochemical properties of T-Nb_2_O_5_/RMF show a trend of 1:6 > 1:10 > RMF > 1:2 > 1:1, which proves that the appropriate amount of T-Nb_2_O_5_ loading plays an important role in the electrochemical properties of the composites. The introduction of excessive metal oxides leads to the blockage of the pores of RMF itself, which seriously reduces the migration rate of lithium ions and deteriorates the wettability of the electrolyte, resulting in a significant reduction of the capacity, while the introduction of too little metal oxides results in less active material and a decrease in the lithium storage capacity. Therefore, the best electrochemical properties were obtained at the ratio 1:6, exhibiting an initial capacity of 1320.8 mA h g^−1^ at 0.1 C and subsequently stabilizing at 945.2 mA h g^−1^ after 100 cycles. Figure 5b shows that when charging and discharging at a high current density of 1 C, the initial specific capacity of 332.8 mA h g^−1^ is obtained. The specific capacity of 207.2 mA h g^−1^ is still maintained after 650 cycles, with a decay rate of 0.058% per cycle, which shows good cyclability. In addition, the material also exhibits good rate performance, as shown in Figure 5c. The discharge-specific capacities are found to be 663.3 mA h g^−1^, 463.5 mA h g^−1^, 379.6 mA h g^−1^, 291.3 mA h g^−1^, 228.1 mA h g^−1^, and 181.5 mA h g^−1^ at 0.1 C, 0.2 C, 0.5 C, 1 C, 2 C and 5 C current densities, respectively. When the current density drops from 5 C to 0.1 C, the battery shows a great recovery capacity of 817.6 mA h g^−1^, which is even better than the initial discharge specific capacity. This may be due to the fact that with the increase of the cycling time, the active material of the electrode can be fully contacted with the electrolyte to form a thicker and more stable SEI film. The SEI film effectively improves the capacity of the composite material of the deep-embedded lithium and the diffusion rate of lithium ions inside the electrode. Figure 5d shows the capacity–voltage curves of T-Nb_2_O_5_/RMF 1:6 composites at different current rates. The result demonstrates that the batteries assembled with these materials can effectively adjust their reaction rates to accommodate the accelerated charge transfer rates associated with variations in charge and discharge current densities. From Table 2, by comparing the electrochemical properties with other anode materials prepared in the literature for dual-ion batteries, it can be seen that the T-Nb_2_O_5_/RMF composites prepared in this work have a very high specific capacity at low current densities, which provides a great advantage for the preparation of high-performance dual-ion batteries.

**Figure 6:**
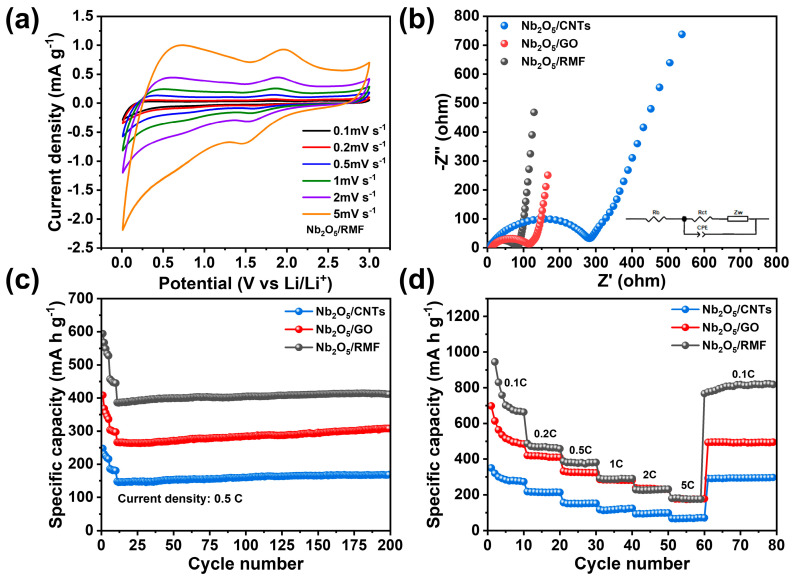
(**a**) CV curves of T-Nb_2_O_5_/RMF composite at different scanning rates (**b**) EIS curves of different T-Nb_2_O_5_/C composites (**c**) cycle performance of different T-Nb_2_O_5_/C composites (**d**) rate performance of different T-Nb_2_O_5_/C composites.

The CV curves of T-Nb_2_O_5_/RMF 1:6 were tested in the range of 0.01–3 V at a scan rate of 0.1–5 mV s^−1^, as illustrated in Figure 6a. It is observed that the cathodic peaks and anodic peaks at 1.48 V and 1.98 V, respectively, correlated with the lithium embedding and delithiation behaviors of T-Nb_2_O_5_ during electrochemical processes. This indicates that the composites exhibit a highly reversible lithium storage process. Figure 6b presents the AC impedance spectra and the fitted equivalent circuit diagrams for T-Nb_2_O_5_ loaded on carbon substrates of different dimensions. As shown in Figure 6b, all three composite materials exhibit a semicircular feature in the high-frequency region and a linear feature in the low-frequency region. The resistance between the electrode and the electrolyte, known as the solution resistance (R_b_), is reflected by the intersection point between the EIS curve and the x-axis. The high-frequency semicircle corresponds to the charge transfer resistance (R_ct_), while the low-frequency linear portion is associated with the Warburg impedance (Z_w_), which reflects the diffusion of lithium ions in the active anode material. Among the three materials, Nb_2_O_5_/RMF shows the smallest impedance, while Nb_2_O_5_/CNTs shows the largest, further confirming the performance differences among the materials. Figure 6c shows a comparison of the cycling performance of the three composites at 0.5 C. The cells were run for 5 revolutions at 0.1 C and 0.2 C, respectively, and then elevated to the level of 0.5 C to promote the formation of the SEI film. Hence, the sudden drop is formed in the front part of the curve. According to the curves, it can be seen that T-Nb_2_O_5_/RMF has the best cycling performance of 411.4 mA h g^−1^ in 200 cycles. Figure 6d shows the comparison of the rate performance of the three composites. At 0.1 C, the specific capacity of T-Nb_2_O_5_/RMF was as high as 702.4 mA h g^−1^, which was the highest among the three, followed by Nb_2_O_5_/GO, and Nb_2_O_5_/CNTs was the lowest. With the increase of the current density, the rate of Nb_2_O_5_/GO exhibits the least capacity weakening trend, and even the specific capacity is comparable to that of Nb_2_O_5_/RMF at high current densities, which is attributed to the extremely high electrical conductivity and electron mobility of graphene. All three materials showed good capacity recovery when the current density recovered from 5 C to 0.1 C. The highly developed pore structure and large specific surface area of Nb_2_O_5_/RMF provided enough space for contact between the active ingredient and the electrolyte. The three-dimensional reticulated carbon skeleton gives the composite material excellent structural stability, and the introduced T-Nb_2_O_5_ nanoparticles are firmly anchored within the pores of the material. The composite material has a good degree of surface defects and a well-connected carbon skeleton, which offers a more convenient path for the conduction of lithium ions and charge and accelerates the overall reaction kinetics of the battery. Therefore, among the three kinds of spatial structures, the three-dimensional mesh carbon skeleton structure has a better nanoparticle composite effect and excellent electrochemical performance, which has a good prospect for the application of dual-ion battery electrode materials.

**Figure 7:**
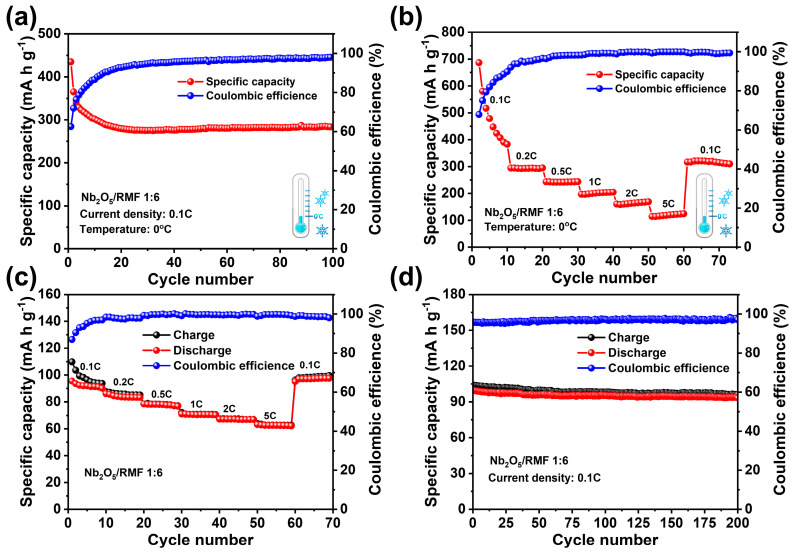
(**a**) Cycling performance curve at 0.1 C of battery assembled with T-Nb_2_O_5_/RMF 1:6 composite in 0 °C low-temperature environment (**b**) rate performance curve of battery assembled with T-Nb_2_O_5_/RMF 1:6 composite in 0 °C low-temperature environment (**c**) Cycling performance curve at 0.1 C of dual-ion battery with T-Nb_2_O_5_/RMF 1:6 as anode and graphite as cathode (**d**) rate performance curve of dual-ion battery with T-Nb_2_O_5_/RMF 1:6 as anode and graphite as cathode.

In order to cope with the various conditions of practical applications, this work also carried out low-temperature electrochemical performance tests at 0 °C. T-Nb_2_O_5_/RMF 1:6 composites were selected as the electrode materials to be assembled into a half cell, which had previously shown the best performance. As shown in Figure 7a,b, the composite material can achieve an initial discharge specific capacity of 435 mA h g^−1^ at 0.1 C, and after 100 cycles, the specific capacity can be stabilized at 283.6 mA h g^−1^. At various current densities from 0.1 to 5 C, the composite material can achieve 383.2 mA h g^−1^, 293.2 mA h g^−1^, 242.5 mA h g^−1^, 203.6 mA h g^−1^, 167. 6 mA h g^−1^, 123.3 mA h g^−1^, respectively. The specific capacity gradually stabilizes at 311.2 mA h g^−1^ when the current density is returned to 0.1 C. The specific capacity did not increase greatly when the specific capacity was restored from high to low current density at low temperatures, suggesting that temperature may affect the electrochemical behavior of the materials and the formation of SEI films to some extent. This indicates that the composite electrodes can still achieve stable cycling performance and excellent rate performance even at low ambient temperatures.

In this work, the T-Nb_2_O_5_/RMF 1:6 composite was also used as the anode material of the dual-ion battery full cell, matched with graphite cathode, and the full-cell performance was tested, in which the voltage range of the test was 3–5 V. The full-cell cycling performance curve at 0.1 C is shown in Figure 7c. It shows that the initial capacity of this dual-ion full-cell battery at 0.1 C was 99.6 mA h g^−1^. The capacity stability was 93.3 mA h g^−1^ after 200 cycles, with a capacity decay rate of 0.031% per cycle, which shows the dual-ion battery has excellent cycling stability. Figure 7d shows the rate performance of the full cell at 0.1 C to 5 C. The specific capacities at different current density platforms are 93.8 mA h g^−1^, 83.3 mA h g^−1^, 77.3 mA h g^−1^, 70.5 mA h g^−1^, 66.9 mA h g^−1^, and 62.4 mA h g^−1^, respectively. The specific capacity is 97.2 mA h g^−1^ when getting back to 0.1 C; the overall capacity change trend is not obvious with the change of current density, indicating that the battery has excellent rate capability. Figure S6 shows the results of AC impedance testing of a dual-ion battery with T-Nb_2_O_5_/RMF 1:6 as the anode material and graphite as the cathode material before and after 50 charge/discharge cycles. The result indicates a noticeable increase in the radius of the semicircle in the high-frequency region and a change in the slope of the line in the low-frequency region, suggesting that both the charge transfer resistance and lithium-ion diffusion resistance within the full-cell battery increase after cycling. This effect is attributed to the irreversible phase transformation of the electrode material during charge/discharge cycles, leading to a gradual decline in battery capacity over time. This is related to the irreversible phase transition of the electrode material in the charge/discharge cycle so that the battery will have a certain capacity decay in the cycling process.

**Figure 8:**
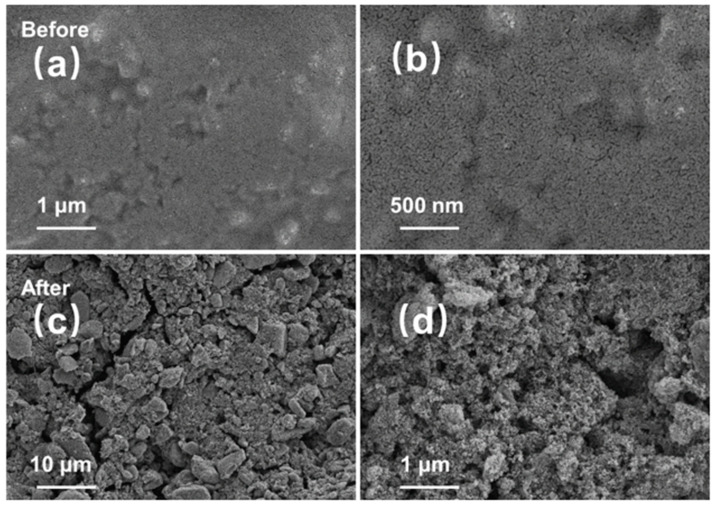
SEM images of electrodes coated with T-Nb_2_O_5_/RMF composites, (**a**,**b**) before cycling, (**c**,**d**) after cycling.

In order to clearly observe the structural stability of T-Nb_2_O_5_/RMF composites during charge/discharge cycling, the electrode sheets coated with T-Nb_2_O_5_/RMF composites before and after cycling were characterized by SEM images from Figure 8. It can be seen that the surface of the electrode is relatively flat and the pore structure on the surface can be observed after magnification in Figure 8a,b. Compared with the SEM image of the surface of the electrode pole piece after cycling in Figure 8c, the amorphous porous carbon structure of the composite was not obviously damaged, in the porous structure of the material can still be clearly observed from the magnified Figure 8d. The T-Nb_2_O_5_ nanoparticles are still uniformly dispersed in the pore channels, which effectively avoids the phenomenon of pulverization of the metal material. Meanwhile, the large specific surface area provided by the porous structure is conducive to the contact of the active substance with the electrolyte, which ensures the structural stability of the composite material and improves the transport rate of lithium ions at the electrode material–electrolyte interface.

## 3. Materials and Methods

Ammonium niobate oxalate hydrate was purchased from Shanghai Dibber Chemical Technology Co., Ltd. (Shanghai, China), resorcinol, melamine, formaldehyde and sodium hydroxide were purchased from Sinopharm Chemical Reagent Co., Ltd. (Shanghai, China), and silica sol was purchased from Shanghai McLean Biochemical Technology Co. (Shanghai, China). Graphene oxide solution and carbon nanotubes were purchased from Shanxi Institute of Coal Chemistry, Chinese Academy of Sciences (Taiyuan, China); hydrochloric acid solution and N-methylpyrrolidone were purchased from Shanghai Taitan Science and Technology Co Ltd. (Shanghai, China); polypropylene (PP) diaphragm was purchased from Celgard Corporation (Concord, NC, USA); and polyvinylidene fluoride was purchased from Arkema France (Paris, France).

The hard template method for the preparation of nitrogen-doped mesoporous carbon materials (RMF) was carried out as follows: in 20 mL of deionized water, 4.85 g of resorcinol and 7.15 g of formaldehyde solution were added and dissolved with stirring in a water bath at 40 °C to obtain the RF solution. In 20 mL of deionized water, 5.56 g of melamine and 10.73 g of formaldehyde were added again and dissolved with stirring in a water bath at 70 °C, then cooled to room temperature to obtain the MF solution. The RF solution was mixed with the MF solution, and 70 g of SM-30 (silica sol) was added, and then mixed, stirred again for 5 min at room temperature, poured into a plastic bottle, and then sealed and reacted at 80 °C for 3 days, after which it was freeze-dried to obtain the nitrogen-doped mesoporous carbon precursor containing silicon.

The precursor was placed in a high-temperature tube furnace with nitrogen venting at a rate of 2 °C min^−1^ to 400 °C for 1 h and then continued to 800 °C for 3 h. The precursor was then cooled down to room temperature naturally. The carbonized samples were placed in plastic bottles and etched with 2 mol L^−1^ NaOH solution for about 3 days, during which time the etched supernatant was removed and replaced with a freshly prepared 2 mol L^−1^ NaOH solution to ensure adequate removal of the SiO_2_ template. After the etching, the sample was washed several times with deionized water and ethanol until neutral pH. Subsequently, it was freeze-dried in a lyophilizer to obtain nitrogen-doped mesoporous carbon materials (RMFs) with well-developed pore structures, which were abbreviated as RMFs because they were prepared from resorcinol melamine-formaldehyde.

In order to reach Nb/C mass ratios of 1:1, 1:2, 1:6, and 1:10, 0.6 g, 0.3 g, 0.1 g, and 0.06 g of niobium ammonium oxalate hydrate were added to 10 mL of deionized water and stirred thoroughly for 30 min. After that, 0.4 g of the previously prepared nitrogen-doped mesoporous carbon material (RMF) was dispersed in the above aqueous solution and ultrasonicated for 20 min. The resulting mixture was stirred magnetically in a water bath heated at 60 °C for 8 h and then washed and freeze-dried. After drying, the samples were carbonized in a tube furnace under nitrogen ventilation at a temperature of 700 °C and a residence time of 3 h. The T-Nb_2_O_5_/RMF composites were finally obtained.

## 4. Conclusions

In summary, nitrogen-containing mesoporous carbon materials were synthesized by hard template method using resorcinol, melamine and formaldehyde solutions as raw materials. The prepared composites present a three-dimensional reticulated skeleton structure with excellent stability and a highly developed pore structure, which facilitates the diffusion and transfer of lithium ions on the electrode surface. The nitrogen-doped porous carbon and T-Nb_2_O_5_ show good synergistic effects, and the Nb_2_O_5_ nanoparticles can not only effectively shorten the ion/electron transport distance in RMF but also alleviate the strain and stress caused by the volume change in the lithiation/delithiation process. The doping of nitrogen and the introduction of T-Nb_2_O_5_ enhance the surface polarity of the composites by destroying the uniform electronic arrangement on the surface of the carbon material, making it possess more active sites and accelerating the electrode reaction rate. The synergistic effect of nitrogen doping with carbon substrate can further improve the electronic conductivity and pseudocapacitive behavior of the active materials. The composite material exhibits a high specific capacity of 945.2 mA h g^−1^ at 0.1 C. At a high current density of 1 C, the initial specific capacity of the battery assembled with the T-Nb_2_O_5_/RMF composite material is 332.8 mA h g^−1^. The specific capacity of 207.2 mA h g^−1^ is still retained after 650 cycles, with a decay rate of 0.058% per cycle, which is very excellent in cycle stability. At a low temperature of 0 °C, the battery still achieves an initial discharge specific capacity of 435 mA h g^−1^ at 0.1 C, which is stabilized at 283.6 mA h g^−1^ after 100 cycles, and a specific capacity of 123.3 mA h g^−1^ at a high current density of 5 C, which recovers to 311.2 mA h g^−1^ when the current density returns to 0.1 C. The initial specific capacity of the full-cell dual-ion battery assembled with graphite cathode was 99.6 mA h g^−1^ at 0.1 C, and the capacity stabilized at 93.3 mA h g^−1^ after 200 cycles with a capacity decay rate of 0.031% per cycle. Good low-temperature performance and full-cell performance also open up possibilities for practical battery applications. This composite material provides a feasible idea for the synthetics of the anode of dual-ion batteries.

## Figures and Tables

**Table 1:** Porosity parameters of T-Nb_2_O_5_/C composite and RMF.

Samples	S_BET_ (m^2^/g)	V_t_ (cm^3^/g)	D_meso_ (nm)
RMF	1008.6	4.51	15.13
T-Nb_2_O_5_/RMF	1568.5	2.79	7.56
T-Nb_2_O_5_/GO	263.9	0.61	4.11
T-Nb_2_O_5_/CNTs	135.3	0.40	3.78

**Table 2:** Comparison of electrochemical performances of different dual-ion battery anodes.

Electrolyte	Specific Capacity	Reference
T-Nb_2_O_5_/RMF	945 mA h g^−1^ after 100 cycles at 100 mA g^−1^	This work
Co_3_O_4_/carbon fiber paper (CFP)	488 mA h g^−1^ after 40 cycles at 200 mA g^−1^	[43]
MoSe_2_/nitrogen-doped carbon (NC)	759 mA h g^−1^ after 100 cycles at 100 mA g^−1^	[44]
MoO_2_/carbon matrix	734 mA h g^−1^ after 350 cycles at 50 mA g^−1^	[45]
SnO_2_	613 mA h g^−1^ after 60 cycles at 60 mA g^−1^	[46]
Li_6_C_12_O_12_	730 mA h g^−1^ after 100 cycles at 210 mA g^−1^	[47]
Sb_2_O_3_@MCNF	626.9 mA h g^−1^ after 80 cycles at 100 mA g^−1^	[42]

## Data Availability

Data are contained within the article and Supplementary Materials.

## Supplementary Materials

The following are the Supporting Information to this article: https://www.mdpi.com/article/10.3390/molecules30020227/s1, The experimental part is described in detail, including the preparation methods of Nb_2_O_5_/GO, and Nb_2_O_5_/CNTs; instrumental parameters used for SEM, TEM, XRD, RAMAN, BET, and XPS characterization; electrode sheet preparation for assembling the button cell, and slurry ratios; instrumental parameters used for CV, EIS, and electrochemical performance testing; Nb_2_O_5_/GO and Nb_2_O_5_/CNTs; SEM and TEM plots; XRD and RAMAN spectra of Nb_2_O_5_/RMF, Nb_2_O_5_/GO, and Nb_2_O_5_/CNTs; weightlessness analysis curves of different ratios of Nb_2_O_5_/RMF in air atmosphere; XPS spectra of Nb_2_O_5_/GO and Nb_2_O_5_/CNTs; EIS curves before and after 50 cycles of dual-ion battery full cell with T-Nb_2_O_5_/RMF 1:6 as anode and graphite as cathode. Figure S1 (a,b) SEM images and (c–f) TEM images of T-Nb_2_O_5_/GO composites. Figure S2 (a,b) SEM images and (c–f) TEM images of T-Nb_2_O_5_/CNTs composites. Figure S3 (a) XRD pattern (b) Raman pattern of different T-Nb_2_O_5_/C composites. Figure S4 (a) TG curves and (b) the thermogravimetric rate curves of RMF and different proportions of T-Nb_2_O_5_/RMF composites in air atmosphere. Figure S5 (a–d) XPS spectrum of T-Nb_2_O_5_/GO composite. (e–f) XPS spectrum of T-Nb_2_O_5_/CNTs composite. Figure S6 EIS curves before and after 50 cycles of dual-ion battery full cell with T-Nb_2_O_5_/RMF 1:6 as anode and graphite as cathode.
