# Supplementary material for: Enhanced Electrochemical Performance of Dual-Ion Batteries with T-Nb2O5/Nitrogen-Doped Three-Dimensional Porous Carbon Composites
Source: Molecules. 2025 Jan 8;30(2):227. doi: 10.3390/molecules30020227 (PMC11767531; doi:10.3390/molecules30020227)
Supplement: Supplementary file 1 [file molecules-30-00227-s001.zip › molecules-3410011-supplementary.pdf]

Supporting Information

Enhanced Electrochemical Performance of Dual-Ion Batteries with T-Nb_2O_5/Nitrogen-Doped Three-Dimensional Porous Carbon Composites

Chen Qi [1], Duo Ying [1], Cheng Ma [1], Wenming Qiao [1], Jitong Wang [1,2,*] and Licheng Ling [1]

[1] State Key Laboratory of Chemical Engineering, East China University of Science and Technology, Shanghai 200237, China; 18919095863@163.com (C.Q.); shapeofvoice@163.com (D.Y.); mac@ecust.edu.cn (C.M.); qiaowm@ecust.edu.cn (W.Q.); lchling@ecust.edu.cn (L.L.)

[2] Guangxi Key Laboratory of Petrochemical Resource Processing and Process Intensification Technology, School of Chemistry and Chemical Engineering, Guangxi University, Nanning 530004, China

* Correspondence: wangjt@ecust.edu.cn

Experimental

S1 Synthesis of Nb_2O_5/carbon nanocomposites

S1.1 Nb_2O_5/GO

First, 0.2 g of polyether P123 was dissolved in 20 mL of deionised water, and then 20 mL of a 5 mg mL^{-1} rGO solution was added to the solution with stirring. The resulting P123/GO solution was sonicated for 20 min. On the other hand, a certain mass of ammonium niobate oxalate hydrate was dissolved in 20 mL of deionised water. The ammonium niobate oxalate solution was then added to the P123/rGO solution along with 5 drops of 2 M HCl solution under magnetic stirring to form a homogeneous solution. The solution was poured into a 100 mL autoclave reactor with a PTFE liner and then heated at 180 °C for 24 hours. Subsequently, the product obtained by filtration was collected, washed alternately with deionised water and ethanol several times, and finally freeze-dried to obtain the uncarbonated T-Nb_2O_5/GO composite. Finally, the freeze-dried samples were carbonised at 700 °C for 3 h under constant nitrogen flow at a temperature increase rate of 2 °C min-1 to obtain T-Nb_2O_5/GO composites. The ratio of T-Nb_2O_5 to carbon was regulated by varying the amount of ammonium niobate oxalate hydrate.

S1.2 Nb_2O_5/CNTs

A certain mass of ammonium niobate oxalate hydrate was weighed and added into 20 mL of deionised water and stirred thoroughly. At the same time, 0.1 g of CNTs was dispersed in 20 mL of deionised water and ultrasonicated in an ultrasonic cleaner for 20 min to form a homogeneous solution. The ammonium niobate oxalate solution was mixed with the aqueous dispersion of CNTs, and 5 drops of 2 M HCl were added, and the homogeneous solution was poured into a 100 mL stainless steel kettle lined with PTFE, and the reaction was set at a high temperature and high pressure for 24 hours at 180 °C. The product was then filtered, and the reaction was carried out at a high

temperature and high pressure. The reaction product was then filtered and washed with deionised water and ethanol for several times before freeze-drying. The freeze-dried powder samples were carbonised in a tube furnace under nitrogen atmosphere at 700 ^{o}C with a residence time of 3 h to obtain T-Nb_2O_5/CNTs composites.

S2 Characterization

The field emission scanning electron microscope (SEM) instrument used in this experiment was a ZEISS Sigma 300 from Baden-Württemberg, Germany.

The transmission electron microscope (TEM) equipment used in this experiment is FEI Talos F200X G2 from Massachusetts, America.

The XRD test equipment for this experiment was an X-ray diffractometer model Rigaku D/mAh X 2550 from Tokyo, Japan, with diffraction angle 2θ = (10°, 80°) with a Cu-Kα diffraction source (λ = 1.5406 Å).

The XPS equipment used in this experiment was an X-ray photoelectron spectrometer from Thermofisher Nexsa (Massachusetts, USA), to analyse the composition of the samples and the bonding energies, etc. The XPS ray source was Al/ Kα, and the operating voltage was set to 12 kV.

The model of the equipment used for the BET test was Quadrasorb SI-MP-9 automatic nitrogen adsorption and desorption instrument (Florida, USA). The pore size distribution curve of the material was based on the DFT/BJH method, the calculation of specific surface area was based on the BET method, and the calculation of total pore volume of the material was based on the nitrogen adsorption at the AD point, and the QuadraWin software was used for data processing.

The Raman instrument used in this experiment was a Renishaw in Via Reflex Raman spectrometer (Tokyo, Japan), and the laser wavelength used for irradiation was selected to be 532 nm.

S3 Electrode Fabrication

In this work, coating method was used to prepare negative electrode sheets. Firstly, the desired active substance, conductive carbon black (super-C) and polyvinylidene fluoride binder (PVDF) were mixed in the ratio of 8:1:1, and then mixed and stirred with an oily solvent, N-methylpyrrolidone (NMP), until a homogeneous active slurry was obtained. Next, the prepared reactive slurry was uniformly applied to the smooth copper foil and then vacuum dried at 80 °C for about 12 hours, followed by cutting. It was cut into round negative electrode sheets of 12 mm diameter using a sheet punching machine and weighed.

In the performance test of the dual-ion battery full cell, commercial graphite was chosen as the electrode material for the positive electrode in order to test and evaluate the electrochemical performance of the T-Nb_2O_5/carbon composite in the full cell. Graphite, conductive agent super C and PVDF were firstly mixed in the mass ratio of 8:1:1, and an appropriate amount of N-methylpyrrolidone (NMP) was gradually added as a solvent to stir and dissolve during the mixing process. After prolonged mixing, a homogeneous slurry of suitable viscosity was formed and coated on the aluminium foil collector, and then placed in an oven at 50 °C for vacuum drying. The coated and dried aluminium foil was cut into 12 mm diameter graphite cathode wafers under a punching machine.

S4 Electrochemical measurements

All electrochemical performance of cell was performed by using CR2025 coin-type cells in glove box (Ar gas). The electrolyte system was selected as 1M $LiPF_6$ in EC-DMC-EMC (1:1:1, vol %) and the diaphragm system was selected as polypropylene (PP) diaphragm.

In this experiment, the constant current charging and discharging test programme is based on the LAND CT3001A battery test system (version number:V7.4), and the

cycle current density is determined from the mass of the active substance and the cycle multiplication rate, with the setting of 1 C=1A g^{-1} and the voltage range of 0.01-3 V. The battery is then charged and discharged by the LAND CT3001A battery test system. The specific capacities (C, mAh g^{-1}) of both half-cells and full cells were derived from GCD curves according to the equation : $C=\frac{I\times t}{m}$, in which I is the discharge current, t is the discharge time, and m is the total mass loading。

In this experiment, cyclic voltammetry (CV) was performed on a Beijing Chenhua CHI600E electrochemical workstation (Beijing, China), and the voltage range of the test was selected as 0.01-3 V. The different scanning rates were set to be 0.1 mV/s, 0.2 mV/s, 0.5 mV/s, 1 mV/s and 2 mV/s, respectively.

In this work, the AC impedance method was performed on a Gamary Reference 600+ electrochemical workstation (Warminster, USA) with a frequency range of 100 kHz~0.01 Hz, where the polarisation voltage magnitude was set to 5 mV.

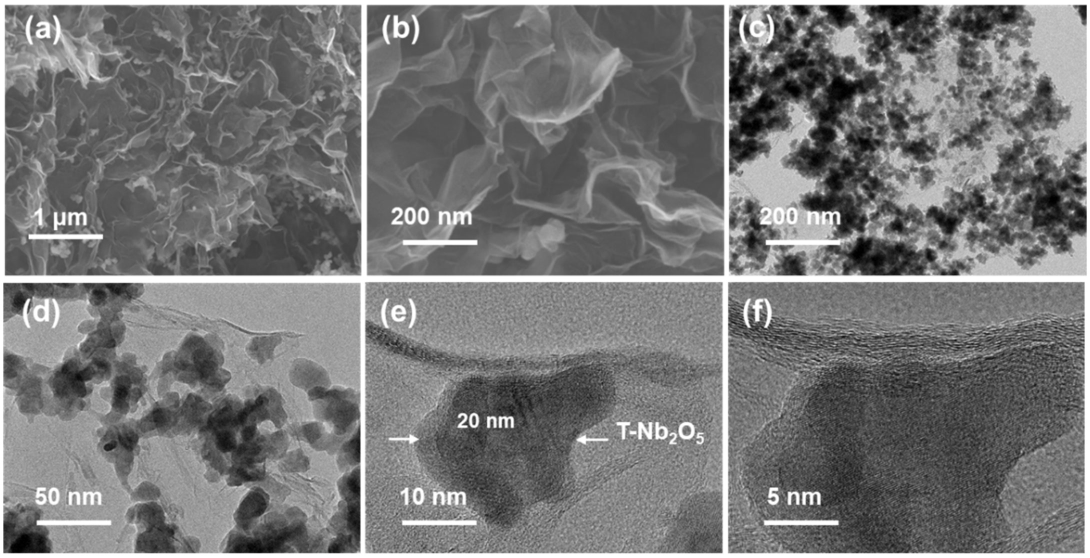

Fig. S1 (a- b) SEM images and (c- f) TEM images of T-Nb_2O_5/GO composites.

The layered structure of graphene and T-Nb_2O_5 nanoparticles dispersed on the surface of graphene can be clearly seen, and the layered structure of graphene has been curled to a certain extent, and even formed a more complex reticular structure.

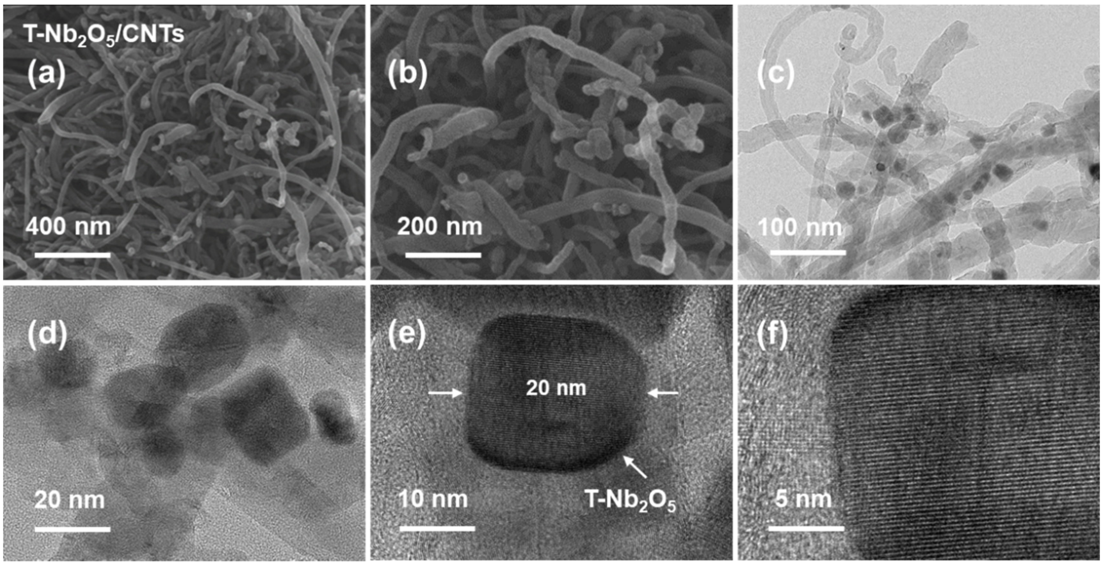

Fig. S2 (a- b) SEM images and (c- f) TEM images of T-Nb_2O_5/CNTs composites.

It can be seen that the carbon nanotubes are irregularly interwoven, while the T-Nb_2O_5 nanoparticles are bound on the surface and inside of the carbon nanotubes.

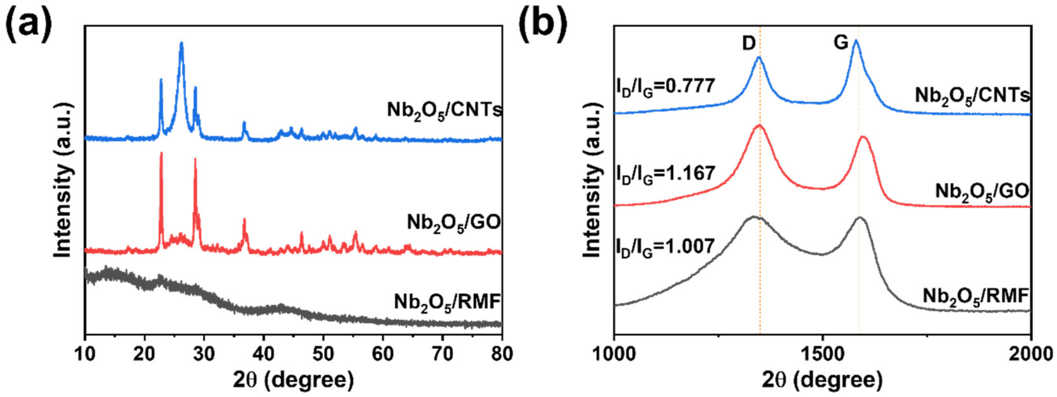

Fig. S3 (a) XRD pattern (b) Raman pattern of different T-Nb_2O_5/C composites.

The 2D T-Nb_2O_5/GO shows the most pronounced T-Nb_2O_5 characteristic peaks, followed by the 1D T-Nb_2O_5/CNTs, while the 3D T-Nb_2O_5/RMFs show broad carbon characteristic peaks. The I_D/I_G of 2D T-Nb_2O_5/GO is the largest, followed by 3D T-Nb_2O_5/RMF, while 1D T-Nb_2O_5/CNTs are the smallest.

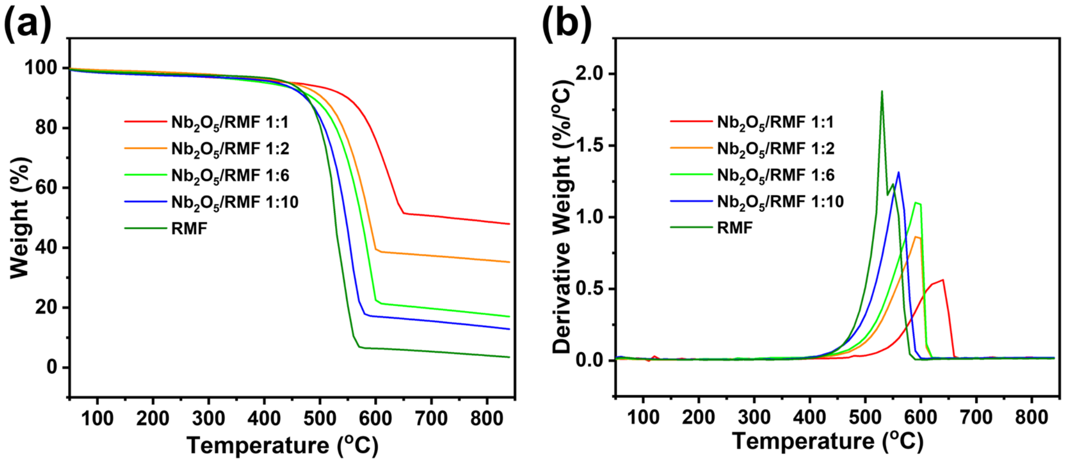

Fig. S4 (a) TG curves and (b) the thermogravimetric rate curves of RMF and different proportions of T-Nb_2O_5/RMF composites in air atmosphere.

With the increase of T-Nb_2O_5 content in the materials, the thermal stability of the composites is enhanced and the pyrolysis temperature is gradually increased.

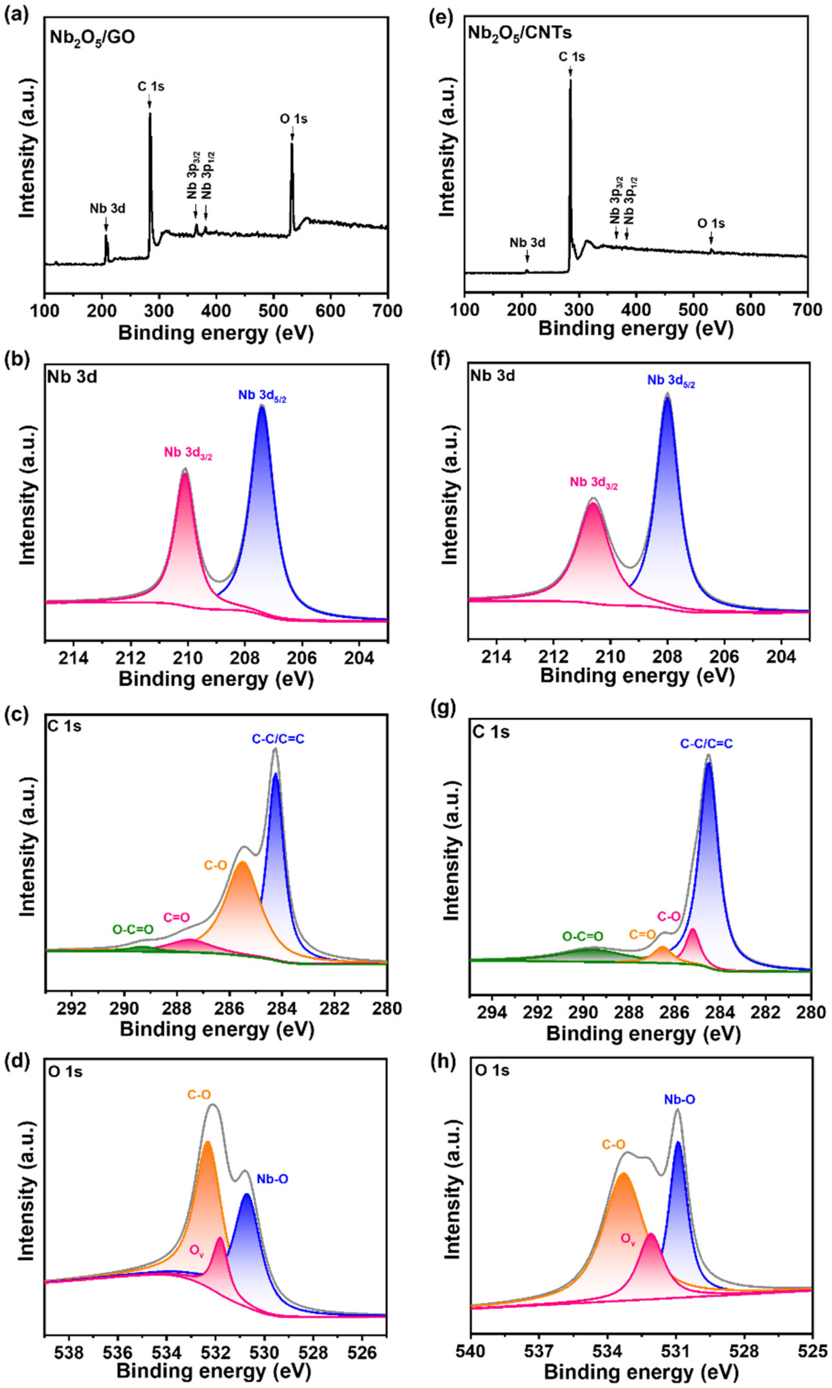

Fig. S5 (a-d) XPS spectrum of T-Nb_2O_5/GO composite. (e-f) XPS spectrum of T-Nb_2O_5/CNTs composite.

T-Nb_2O_5/GO has a stronger O peak, presumably due to the presence of oxygen-containing functional groups in graphene oxide, and also has a higher Nb peak for T-Nb_2O_5/GO, demonstrating that the 2D materials described previously have the highest T-Nb_2O_5 particle loading capacity.

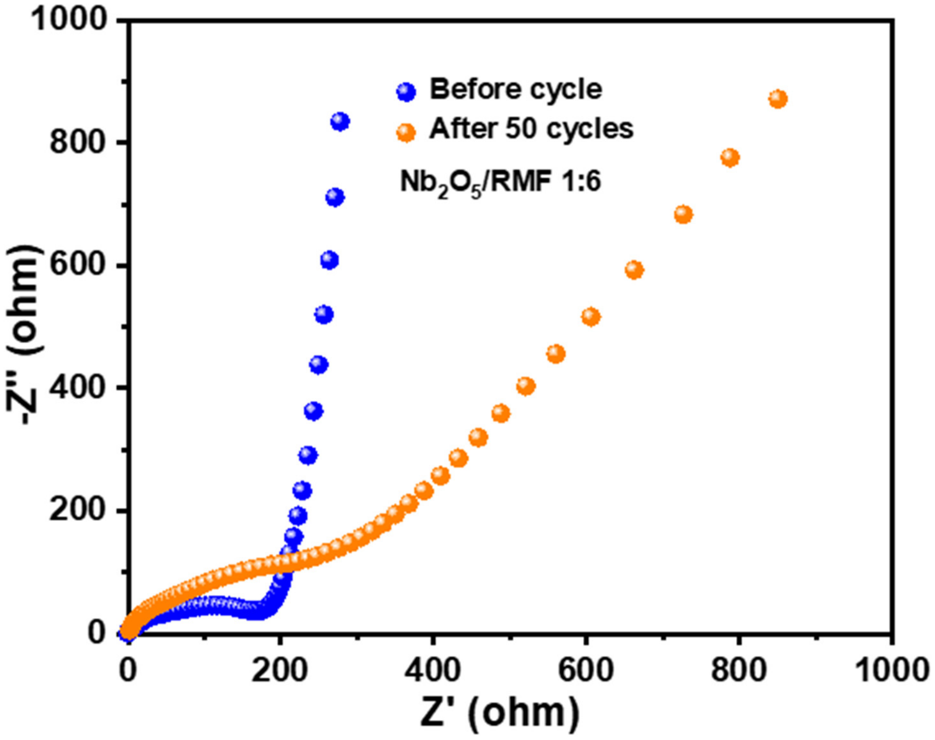

Fig. S6 EIS curves before and after 50 cycles of dual-ion battery full cell with T-Nb_2O_5/RMF 1:6 as anode and graphite as cathode.

The change in impedance before and after cycling is related to the irreversible phase transition of the electrode material during the charging and discharging cycle, and thus the battery will experience a certain degree of capacity degradation during cycling.
